# *N*-(3-Chloro­propion­yl)-*N*′-phenyl­thio­urea

**DOI:** 10.1107/S1600536810005271

**Published:** 2010-02-13

**Authors:** Eliyanti A. Othman, Siti K. C. Soh, Bohari M. Yamin

**Affiliations:** a School of Chemical Sciences and Food Technology, Universiti Kebangsaan Malaysia, UKM 43500 Bangi Selangor, Malaysia

## Abstract

The title compound, C_10_H_11_ClN_2_OS, adopts a *cis*-*trans* configuration with respect to the position of the phenyl and 3-chloro­propionyl groups relative to the thiono group across the C—N bonds. The benzene ring is perpendicular to the propionyl thio­urea fragment with a dihedral angle of 82.62 (10)°. An intra­molecular N—H⋯O inter­action occurs. The crystal structure is stabilized by inter­molecular N—H⋯S hydrogen bonds, which link pairs of mol­ecules, building up *R*_2_^2^(8) ring motifs, and C—H.. π inter­actions.

## Related literature

For related structures, see: Ismail *et al.* (2007 ▶); Ismail & Yamin (2009 ▶). For hydrogen-bond motifs, see: Etter *et al.*(1990 ▶); Bernstein *et al.* (1995 ▶). For reference bond lengths, see: Allen *et al.* (1987 ▶).
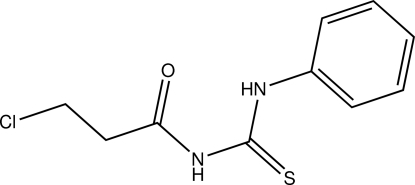


## Experimental

### 

#### Crystal data

C_10_H_11_ClN_2_OS
*M* *_r_* = 242.72
Triclinic, 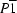

*a* = 5.8088 (12) Å
*b* = 10.467 (2) Å
*c* = 10.660 (2) Å
α = 112.811 (3)°
β = 101.855 (3)°
γ = 95.483 (3)°
*V* = 573.6 (2) Å^3^
*Z* = 2
Mo *K*α radiation
μ = 0.49 mm^−1^
*T* = 298 K
0.49 × 0.45 × 0.27 mm

#### Data collection

Bruker SMART APEX CCD area-detector diffractometer
Absorption correction: multi-scan (*SADABS*; Bruker, 2000 ▶) *T*_min_ = 0.795, *T*_max_ = 0.879
5617 measured reflections
2103 independent reflections
1798 reflections with *I* > 2σ(*I*)
*R*_int_ = 0.016

#### Refinement

*R*[*F* ^2^ > 2σ(*F* ^2^)] = 0.041
*wR*(*F* ^2^) = 0.113
*S* = 1.04
2103 reflections
136 parameters
H-atom parameters constrained
Δρ_max_ = 0.54 e Å^−3^
Δρ_min_ = −0.43 e Å^−3^

### 

Data collection: *SMART* (Bruker, 2000 ▶); cell refinement: *SAINT* (Bruker, 2000 ▶); data reduction: *SAINT*; program(s) used to solve structure: *SHELXS97* (Sheldrick, 2008 ▶); program(s) used to refine structure: *SHELXL97* (Sheldrick, 2008 ▶); molecular graphics: *SHELXTL* (Sheldrick, 2008 ▶); software used to prepare material for publication: *SHELXTL*, *PARST* (Nardelli, 1995 ▶) and *PLATON* (Spek, 2009 ▶).

## Supplementary Material

Crystal structure: contains datablocks global, I. DOI: 10.1107/S1600536810005271/dn2535sup1.cif

Structure factors: contains datablocks I. DOI: 10.1107/S1600536810005271/dn2535Isup2.hkl

Additional supplementary materials:  crystallographic information; 3D view; checkCIF report

## Figures and Tables

**Table 1:** Hydrogen-bond geometry (Å, °) *Cg*1 is the centroid of the C5–C10 ring.

*D*—H⋯*A*	*D*—H	H⋯*A*	*D*⋯*A*	*D*—H⋯*A*
N2—H2*A*⋯O1	0.86	2.01	2.677 (3)	134
N1—H1*A*⋯S1^i^	0.86	2.53	3.3709 (19)	165
C1—H1*C*⋯*Cg*1^ii^	0.97	2.84	3.466	123

Symmetry codes: (i) 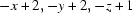; (ii) 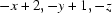.

**Fig. 1.**
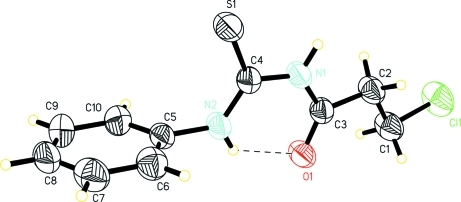
The molecular structure of (I),with displacement ellipsods drawn at the 50% probability level. H atoms are represented as small spheres of arbitrary radii.

**Fig. 2.**
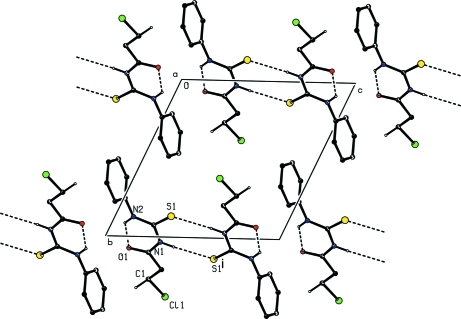
A packing diagram of (I) viewed down the a axis. Hydrogen bonds are shown by dashed lines. H atoms not involved in hydrogen bondings have been omitted for clarity. [Symmetry code: (i) -x+2, -y+2, -z+1]

## supplementary crystallographic information

### Comment

The presence of both alpha and ipso chlorine atoms in 2-chloropropionyl chloride could lead to a complicated reaction when reacted with a nucleophile such as thiocyanate depending on the solvent used. For example, the reaction of 2-chloropropionyl chloride with ammonium thiocyanate and succeedingly with aniline in acetone was found to give 4,5,6-trimethyl-1- phenyl-3,4-dihydropyrimidine-2(1*H*)-thione (Ismail *et al.*, 2007) instead of the expected *N*-phenyl-*N*'-(2-chloropropionyl) thiourea. However, in the present study, the same reaction but with 3-chloropropionyl chloride, the expected *N*-phenyl-*N*'-(3-chloropropionyl)thiourea (I) was indeed obtained.

The whole molecule is not planar (Fig.1). The benzene (C5—C10) ring and propionylthiourea fragment, S1/N1/N2/(C1—C4) are each planar with maximum deviation of 0.062 (2)Å for C3 atom from the least square plane. The benzene ring is roughly perpendicular to the propionylthiourea fragment with dihedral angle between the two planes of 82.62 (10)°. A smaller dihedral angle of 12.68 (7)° was observed in an analogous compound of *N*-(3-chloropropionyl)-*N*'(4-fluorophenyl) thiourea (II) (Ismail & Yamin, 2009).The trans-cis configuration of the propionyl and phenyl groups relative to the thiono group respectively, across their C—N bonds, is maintained. The bond lengths and angles are in normal range (Allen *et al.*,1987) and comparable to those in (II).

There is one intrahydrogen bond, N2—H2A···O1 forming the pseudo-six membered ring, N2/C4/N1/C3/O1···H2A. In the crystal structure, the molecules are linked by N1—H1A···S1 intermolecular hydrogen bond forming dimers through a R_2_^2^(8) ring (Etter *et al.*, 1990 ; Bernstein *et al.*, 1995) extending along the c-axis (Table 1, Fig.2). In addition, there is also a C1—H1C.,.π bond with the benzene (C5—C10) ring (Table 1).

### Experimental

30 ml acetone solution of aniline was added into 30 ml acetone containing 3-chloropropionyl isothiocyanate (1.49, 0.01 mol). The mixture was refluxed for 2 hours. The solution was filtered and left to evaporate at room temperature. The white precipitate obtained after a few days, was washed with water and cold ethanol. The colorless crsytals were obtained by recrystallization from ethanol. Yield 90%; m.p 389.7-391.2 K; ^1^H NMR (400 MHz, CDCl_3_-d_6_):δ (ppm)= 12.32 (s,1*H*), 9.99 (s,1*H*), 7.63 (m,4*H*, H_Ar_), 7.41 (s,1*H*, H_Ar_), 3.8 (d,2*H*,Cl—CH_2_), 2.9 (d,2*H*,CH_2_). ^13^C NMR (400 MHz, CDCl_3_-d_6_):δ (ppm)= 38.32, 39.76, 124.70, 127.35, 127.35, 129.08, 129.08, 137.30, 171.22, 178.49.

### Refinement

After locating the hydrogen atoms from a different-Fourier map, they were positioned geonmetrically with C—H=0.93-0.97Å and N—H=0.86Å respectively, and constrained to all their parent atoms with U_iso_(H)=1.2U_eq_(parent atom). There is a highest peak and deepest hole of 0.97 and 0.81 Å respectively from S1 atom.

### Crystal data

**Table:**

C_10_H_11_ClN_2_OS	*Z* = 2
*M**_r_* = 242.72	*F*(000) = 252
Triclinic, *P*1	*D*_x_ = 1.405 Mg m^−^^3^
Hall symbol: -P 1	Mo *K*α radiation, λ = 0.71073 Å
*a* = 5.8088 (12) Å	Cell parameters from 3405 reflections
*b* = 10.467 (2) Å	θ = 2.1–25.5°
*c* = 10.660 (2) Å	µ = 0.49 mm^−^^1^
α = 112.811 (3)°	*T* = 298 K
β = 101.855 (3)°	Block, colourless
γ = 95.483 (3)°	0.49 × 0.45 × 0.27 mm
*V* = 573.6 (2) Å^3^	

### Data collection

**Table:**

Bruker SMART APEX CCD area-detector diffractometer	2103 independent reflections
Radiation source: fine-focus sealed tube	1798 reflections with *I* > 2σ(*I*)
Detector resolution: 83.66 pixels mm^-1^	*R*_int_ = 0.016
ω scan	θ_max_ = 25.5°, θ_min_ = 2.1°
Absorption correction: multi-scan (*SADABS*; Bruker, 2000)	*h* = −7→7
*T*_min_ = 0.795, *T*_max_ = 0.879	*k* = −12→12
5617 measured reflections	*l* = −12→12

### Refinement

**Table:**

Refinement on *F*^2^	Primary atom site location: structure-invariant direct methods
Least-squares matrix: full	Secondary atom site location: difference Fourier map
*R*[*F*^2^ > 2σ(*F*^2^)] = 0.041	Hydrogen site location: inferred from neighbouring sites
*wR*(*F*^2^) = 0.113	H-atom parameters constrained
*S* = 1.04	*w* = 1/[σ^2^(*F*_o_^2^) + (0.0559*P*)^2^ + 0.2663*P*] where *P* = (*F*_o_^2^ + 2*F*_c_^2^)/3
2103 reflections	(Δ/σ)_max_ < 0.001
136 parameters	Δρ_max_ = 0.54 e Å^−^^3^
0 restraints	Δρ_min_ = −0.43 e Å^−^^3^

### Special details

**Table:**

Geometry. All esds (except the esd in the dihedral angle between two l.s. planes) are estimated using the full covariance matrix. The cell esds are taken into account individually in the estimation of esds in distances, angles and torsion angles; correlations between esds in cell parameters are only used when they are defined by crystal symmetry. An approximate (isotropic) treatment of cell esds is used for estimating esds involving l.s. planes.
Refinement. Refinement of *F*^2^ against ALL reflections. The weighted *R*-factor wR and goodness of fit *S* are based on *F*^2^, conventional *R*-factors *R* are based on *F*, with *F* set to zero for negative *F*^2^. The threshold expression of *F*^2^ > σ(*F*^2^) is used only for calculating *R*-factors(gt) etc. and is not relevant to the choice of reflections for refinement. *R*-factors based on *F*^2^ are statistically about twice as large as those based on *F*, and *R*- factors based on ALL data will be even larger.

### Fractional atomic coordinates and isotropic or equivalent isotropic displacement parameters (Å^2^)

**Table:**

	*x*	*y*	*z*	*U*_iso_*/*U*_eq_	
Cl1	0.23811 (13)	1.37819 (8)	0.50799 (7)	0.0777 (3)	
S1	1.10045 (13)	0.86771 (7)	0.31949 (6)	0.0653 (2)	
O1	0.5152 (3)	1.07380 (16)	0.16725 (14)	0.0539 (4)	
N1	0.7823 (3)	1.03263 (18)	0.32922 (17)	0.0497 (4)	
H1A	0.8413	1.0614	0.4186	0.060*	
N2	0.7847 (3)	0.87565 (18)	0.10659 (17)	0.0484 (4)	
H2A	0.6781	0.9154	0.0753	0.058*	
C1	0.3405 (4)	1.2689 (3)	0.3640 (2)	0.0594 (6)	
H1B	0.2091	1.1935	0.2967	0.071*	
H1C	0.3931	1.3244	0.3167	0.071*	
C2	0.5422 (4)	1.2067 (2)	0.4124 (2)	0.0539 (5)	
H2B	0.4968	1.1623	0.4707	0.065*	
H2C	0.6809	1.2815	0.4696	0.065*	
C3	0.6079 (4)	1.0991 (2)	0.2902 (2)	0.0434 (5)	
C4	0.8769 (4)	0.9252 (2)	0.2443 (2)	0.0452 (5)	
C5	0.8541 (4)	0.7586 (2)	0.00691 (19)	0.0435 (5)	
C6	1.0500 (4)	0.7804 (2)	−0.0412 (2)	0.0543 (5)	
H6A	1.1404	0.8707	−0.0087	0.065*	
C7	1.1101 (5)	0.6653 (3)	−0.1391 (3)	0.0618 (6)	
H7A	1.2421	0.6787	−0.1725	0.074*	
C8	0.9790 (5)	0.5329 (3)	−0.1872 (2)	0.0635 (7)	
H8A	1.0221	0.4565	−0.2524	0.076*	
C9	0.7837 (5)	0.5125 (2)	−0.1393 (3)	0.0646 (7)	
H9A	0.6934	0.4221	−0.1724	0.078*	
C10	0.7203 (4)	0.6258 (2)	−0.0418 (2)	0.0541 (5)	
H10A	0.5872	0.6120	−0.0094	0.065*	

### Atomic displacement parameters (Å^2^)

**Table:**

	*U*^11^	*U*^22^	*U*^33^	*U*^12^	*U*^13^	*U*^23^
Cl1	0.0802 (5)	0.0871 (5)	0.0664 (4)	0.0432 (4)	0.0358 (4)	0.0181 (4)
S1	0.0877 (5)	0.0664 (4)	0.0387 (3)	0.0470 (3)	0.0082 (3)	0.0157 (3)
O1	0.0636 (9)	0.0576 (9)	0.0367 (8)	0.0276 (7)	0.0084 (7)	0.0145 (7)
N1	0.0632 (11)	0.0471 (10)	0.0316 (8)	0.0248 (8)	0.0058 (7)	0.0090 (7)
N2	0.0596 (11)	0.0462 (10)	0.0345 (9)	0.0255 (8)	0.0069 (7)	0.0110 (7)
C1	0.0641 (14)	0.0675 (15)	0.0476 (12)	0.0343 (12)	0.0188 (11)	0.0180 (11)
C2	0.0604 (13)	0.0556 (13)	0.0400 (11)	0.0260 (11)	0.0097 (10)	0.0126 (10)
C3	0.0480 (11)	0.0397 (10)	0.0384 (11)	0.0137 (9)	0.0082 (8)	0.0125 (8)
C4	0.0560 (12)	0.0387 (10)	0.0384 (10)	0.0162 (9)	0.0102 (9)	0.0130 (8)
C5	0.0524 (11)	0.0440 (11)	0.0313 (9)	0.0205 (9)	0.0066 (8)	0.0126 (8)
C6	0.0513 (12)	0.0552 (13)	0.0508 (12)	0.0123 (10)	0.0090 (10)	0.0182 (10)
C7	0.0600 (14)	0.0798 (18)	0.0559 (14)	0.0321 (13)	0.0245 (11)	0.0301 (13)
C8	0.0891 (18)	0.0620 (15)	0.0459 (13)	0.0419 (14)	0.0251 (12)	0.0194 (11)
C9	0.0920 (19)	0.0416 (12)	0.0555 (14)	0.0182 (12)	0.0218 (13)	0.0130 (10)
C10	0.0650 (14)	0.0484 (12)	0.0517 (12)	0.0169 (10)	0.0219 (11)	0.0192 (10)

### Geometric parameters (Å, °)

**Table:**

Cl1—C1	1.778 (2)	C2—H2B	0.9700
S1—C4	1.669 (2)	C2—H2C	0.9700
O1—C3	1.221 (2)	C5—C10	1.369 (3)
N1—C3	1.367 (3)	C5—C6	1.375 (3)
N1—C4	1.386 (3)	C6—C7	1.385 (3)
N1—H1A	0.8600	C6—H6A	0.9300
N2—C4	1.320 (3)	C7—C8	1.361 (4)
N2—C5	1.433 (2)	C7—H7A	0.9300
N2—H2A	0.8600	C8—C9	1.367 (4)
C1—C2	1.490 (3)	C8—H8A	0.9300
C1—H1B	0.9700	C9—C10	1.381 (3)
C1—H1C	0.9700	C9—H9A	0.9300
C2—C3	1.503 (3)	C10—H10A	0.9300
			
C3—N1—C4	128.77 (17)	N2—C4—N1	117.03 (18)
C3—N1—H1A	115.6	N2—C4—S1	123.88 (16)
C4—N1—H1A	115.6	N1—C4—S1	119.09 (15)
C4—N2—C5	122.97 (17)	C10—C5—C6	120.60 (19)
C4—N2—H2A	118.5	C10—C5—N2	119.22 (19)
C5—N2—H2A	118.5	C6—C5—N2	120.17 (19)
C2—C1—Cl1	111.32 (16)	C5—C6—C7	118.7 (2)
C2—C1—H1B	109.4	C5—C6—H6A	120.7
Cl1—C1—H1B	109.4	C7—C6—H6A	120.7
C2—C1—H1C	109.4	C8—C7—C6	121.0 (2)
Cl1—C1—H1C	109.4	C8—C7—H7A	119.5
H1B—C1—H1C	108.0	C6—C7—H7A	119.5
C1—C2—C3	111.71 (17)	C7—C8—C9	119.9 (2)
C1—C2—H2B	109.3	C7—C8—H8A	120.1
C3—C2—H2B	109.3	C9—C8—H8A	120.1
C1—C2—H2C	109.3	C8—C9—C10	120.1 (2)
C3—C2—H2C	109.3	C8—C9—H9A	119.9
H2B—C2—H2C	107.9	C10—C9—H9A	119.9
O1—C3—N1	122.97 (18)	C5—C10—C9	119.7 (2)
O1—C3—C2	123.17 (18)	C5—C10—H10A	120.1
N1—C3—C2	113.86 (17)	C9—C10—H10A	120.1
			
Cl1—C1—C2—C3	172.29 (17)	C4—N2—C5—C6	−86.1 (3)
C4—N1—C3—O1	−3.2 (4)	C10—C5—C6—C7	−0.5 (3)
C4—N1—C3—C2	177.1 (2)	N2—C5—C6—C7	−179.04 (19)
C1—C2—C3—O1	4.3 (3)	C5—C6—C7—C8	−0.1 (3)
C1—C2—C3—N1	−176.1 (2)	C6—C7—C8—C9	0.4 (4)
C5—N2—C4—N1	−175.79 (19)	C7—C8—C9—C10	−0.3 (4)
C5—N2—C4—S1	5.2 (3)	C6—C5—C10—C9	0.6 (3)
C3—N1—C4—N2	−2.1 (3)	N2—C5—C10—C9	179.2 (2)
C3—N1—C4—S1	176.94 (18)	C8—C9—C10—C5	−0.2 (4)
C4—N2—C5—C10	95.3 (3)		

### Hydrogen-bond geometry (Å, °)

**Table:**

Cg1 is the centroid of the C5–C10 ring.

**Table:**

*D*—H···*A*	*D*—H	H···*A*	*D*···*A*	*D*—H···*A*
N2—H2A···O1	0.86	2.01	2.677 (3)	134
N1—H1A···S1^i^	0.86	2.53	3.3709 (19)	165
C1—H1C···Cg1^ii^	0.97	2.84	3.466	123

Symmetry codes:  (i) −*x*+2, −*y*+2, −*z*+1; (ii) −*x*+2, −*y*+1, −*z*.
